# Domestic Summary

**Published:** 1838-07-18

**Authors:** 


### DOMESTIC SUMMARY.

*Mortality of Philadelphia.*—During the week ending 14th July, a week of intense heat, the mortality of Philadelphia has exceeded that of any period, since the epidemic of Asiatic Cholera, in 1832, being greater, by about a hundred deaths, than is usual, at this season, in healthy summers. The mortality has been principally confined to children. Of the two hundred and thirty-one deaths, ninety-four were of children under one year, and a hundred and forty-five, of children under twenty-one years. Fifty-seven deaths are credited to summer complaint, and seventeen to excessive heat.

*Jefferson Medical College of Philadelphia.*—Under the new charter and organization of this institution, all the officers ceased to exist as such, except the old trustees, who were continued by the new charter. It became, therefore, necessary to appoint professors to the vacant chairs. All the former professors were, accordingly, unanimously re-elected.—*Amer. Med. Int.*

*Medical College of Ohio. Dr. Mussey.*—Dr. Mussey, of Dartmouth College, has accepted the professorship of surgery in the Medical College of Ohio, which cannot fail to be largely benefitted by the transfer.

*Medical College of Richmond, Va.*—The trustees of Hampden Sidney College, having organized a medical department in the city of Richmond, Va., lectures, we learn, will commence there on Monday, the 5th of November, 1833, and will be continued until the last week in March.

---

*Trannsylvania Medical School. Dr. N. R. Smith.*—We learn that Dr. N. R. Smith, of Baltimore, has accepted the Chair of Theory and Practice of Physic in this school: but that he will continue to reside in Baltimore except during the winter session.

---

*An account of the use of the Bark of the Slippery Elm Tree (Ulmus fulva) for Bougies, Tents, Catheters, and similar purposes in Surgery.* By William A. McDowell, M. D., of Fincastle, Virginia.

In 1819, the use of slippery elm tents was recommended to my attention by my friend, Dr. John Fleece, of Danville, Ky., as more easily introduced, more pleasantly worn, and better adapted to keep an issue or seton open, than any others. His assertions were amply sustained, by the effects produced in the case of a gentleman whom we there jointly attended.

*Lumbar Abscess.*—From a lumbar, or psoas abscess, the pus had descended to the thigh, and was there lodged, not under the fascia, as is common, but deep-seated, around the bone, of which the periosteum was destroyed. An incision, or puncture, had been made into the abscess, with a bistoury, about the middle of the thigh. This puncture was liable to obstruction, by curdly lumps, that floated in the pus, and such obstruction was always attended with great increase of tension and pain. The case seemed hopeless; but an enlargement of the fistula was obviously proper, and the patient objected to the use of the bistoury. It was in this situation, that the alternative of a slippery elm tent was determined upon.

A strip of the seasoned inner bark, long enough to reach the bone, and to leave an external portion to be bent down over the skin, (by which it could be withdrawn,) smoothly polished, rounded at the point, and shaped as nearly to the size of the puncture as we could get it, was dipped for a few seconds in tepid water. Covered with mucilage, and so slippery that it could with difficulty be held in the fingers, it was introduced into the sinus; where it remained twelve hours, without (according to the patient's report) producing one disagreeable sensation. On its removal, the sinus was found to be enlarged from one-fourth to one-third of an inch, by the expansion the seasoned tent had undergone. Another tent was now prepared, of the size of that just removed in its expanded state, to be next introduced; and so on, in succession, they were used larger and larger, until the opening was made as wide as seemed necessary, without pain, or effusion of blood.

*Mammary Abscess.*—Tents of this description, I have found particularly advantageous in the treatment of mammary abscess.

In cases of deep-seated abscess, but not answering the description of a disease under that name, as given by Mr. Hey, (such I never saw,) but in cases, or more especially in a case, of long standing, with deep sinus, dipping into and through the mamma, with the gland in an indurated, rough, and enlarged condition, I was completely successful, by tenting the different sinuses.

A case of this description had been pronounced cancerous, and I was employed on account of the belief that extirpation must be performed. The tents were made thin, and softened in water, until their flexibility admitted of their following the devious course of the sinus, without the application of force or violence. In six or seven days, the secretion of healthy pus commenced throughout the sinus.

The external openings were enlarged in this, and in most cases, through the skin, with the point of a lancet, previous to introducing tents. After the secretion of healthy pus occurs, the tents should still be made as wide as ever, but a little shorter every day or two, and thinner at the point, and at the sides, that no violence may be done to the granulations.

*Gun-shot Wounds.*—In all abscesses, sinuses, or wounds, that require tenting, the slippery elm merits a preference. To widen a sinus, or to prepare it for the extraction of loose bones during the process of exploration, their superiority is obvious.

A young man of this town, walking behind a companion whilst hunting, and almost in contact with the muzzle of the gun, received the contents of his musket, shot and wadding, (accidentally discharged,) in his shoulder. About an inch of the centre of the clavicle was shattered, and the fragments carried deep into the wound. Some of the shot passed out through the scapula, and some posterior to and below the middle of its basis; the rest, together with most of the wadding, (which was of dry leaves,) and portions of the clavicle, lodged against its flat side. This wound was tented, with broad and thin tents, to keep the opening wide, yet not to prevent discharge, until all the foreign matters were removed with slender forceps, and until the wound healed from the bottom outward.

*Strictures.*—Jan. 13, 1831, I visited Mr. C——, at his father's, in this county, near Haweytown. I found him shut up in a stove room, heated to about 100 degrees, and rolled in blankets, yet tormented with rigors; his emaciation extreme, complexion chlorotic, tongue furred, and countenance unhappy. Mr. C——, (now Dr. C——, of Indiana,) had some weeks previously returned from North Carolina, where he had been advantageously engaged in gold-mining, until he was interrupted by the violence of the disease. It had been gradually increasing for more than three years; within which time, he had been under the management of several physicians—some of them eminent. They afforded him none, or but very transient, relief. From his mines he visited Charleston, and several intermediate places, in quest of surgical aid; but deriving no benefit, he returned to the mines in despair, sold them, and relinquishing all his golden expectations, came back to Bath-Court, to die among his kindred. On arriving at his father's, he put himself under the care of a physician, whom I conceive to be inferior in professional ability to but few in Virginia. After enduring his management for about four weeks, Mr. C—— abruptly dismissed him and sent for me. He said Dr. —— had cauterized him nearly to death, and in despite, too, of his repeated protestations, that this plan had been tried over and over before, and had failed; and he even shuddered as he spoke of his sufferings. The stilet, I learned, had also been used with no better effect.

I inserted an ordinary bougie, of which he had a variety, and at the distance of about three inches, met with a stricture opposing firm resistance: the largest bougie it would admit was smaller than a crow's quill. Not knowing what exactly to do for the stricture, I did nothing. For his general health, I prescribed a purgative of salts and magnesia, and a blue pill of five grains, to be taken daily; a hip-bath occasionally; and a gradual reduction of the temperature of his chamber; leaving him with a promise to visit him again in a few days.

In the course of my reflections on the case, it occurred to me, that one of those expanding slippery elm tents, might be shaped into a bougie, that would expand and cure this stricture; and I forthwith rode out and made the trial. A piece of dried inner bark, shaped to the size of a bougie that would pass the stricture, and slightly tapered, was, after remaining a few seconds in tepid water, passed through the stricture; pushed firmly into it; and suffered to remain four hours. It produced less uneasiness than was anticipated: of pain there was none, and the uneasiness seemed to be overbalanced by the mental relief arising from the contemplation of a new project. On removing the bougie, which required a severe pull, a perfect impression of a stricture, from a fourth to the third of an inch broad, was indented around it; rough, and pitted on one side, as if it had been impressed with a thimble. The bougie had expanded considerably, except that portion of it which was embraced by the stricture. There it appeared but little enlarged, and the bulge beyond had caused the difficulty of extraction. I now prescribed the introduction of such a bougie, every day or two, to be retained as long as it could be tolerated; a blue pill to be taken every third day, and the bowels to be kept open with saline purgatives. The dilatation was at first very gradual; but in ten or twelve days, so large a bougie was required, that it became necessary to double the bark, by gluing the flat sides of two pieces together. On the 17th February he was dismissed cured, and has never since been troubled with the disease.

The slow progress (when compared with any other case that I have since encountered) and difficulty, in the dilatation of this stricture, I have ascribed to the oft-repeated incisions with the stilet, and the ulcerations from the caustic, having converted its inner surface into a cicatrix, with diminished expansibility.

The bougie here recommended, and brought by this case directly in comparison with the caustic and the stilet, it must be evident to any one acquainted with these diseases, is not calculated to supersede either of them; and I recommend it only as an additional agent in treating strictures; yet, in a great majority of cases, it is decidedly superior to either, or to any other plan of which I have any knowledge. It is particularly adapted to all strictures, proceeding from spasmodic contraction, or from circular thickening of the membrane, or from callosity, or indurated enlargement of the surrounding corpus spongiosum, or of the prostrate gland.

But in case of obstruction from a thickening of one side of the urethra, projecting as an excrescence into the canal, in the form of a wart, I can conceive nothing short of extirpation to be adequate to effect a cure; and no plan strikes me, as so eligible for effecting this, as the application of caustic. The stilet would be wholly inadequate, and from the expansive bougie, only temporary relief could be anticipated. It is true, that tumours, even warts, may sometimes be removed from the surface of the body by pressure; but it must be long continued and firm, to a degree not to be endured on a structure so delicate as that of the urethra. In cases of total obstruction, from filling up, or obliteration of a considerable length of the urethra, the stilet is, I conceive, without a rival; and we have no alternative to it but incision through the integuments.

*Fistula in Perinæo—Case.*—In October, 1824, I visited a negro boy aged twelve, the property of Mr. M——, of this county. He had several fistulous openings in the perinæum, through which he passed all his urine. There had been considerable sloughing of the part, and that process still continued. On passing a sound into the urethra, I found the part within the scrotum filled with a calculus. I pushed it back, enlarged one of the sinuses, and extracted it. I prescribed a course of treatment calculated to produce present relief, and requested his master to send him to town as soon as he was well enough for farther treatment. He was so much relieved, that he was not sent until the following spring; when, on the 9th May, I made incisions through the callus of the fistulas, from the perinæum to the urethra, and suffered him to pass no urine without a catheter, until the part had healed. When it was found that all his urine flowed by the natural channel, I permitted him to return home; but advised that his urine should still be passed by the catheter for a few weeks; which, unfortunately, was neglected, and a fistulous opening made its appearance again. The disease progressed, and was unattended to, until May, 1834, when he came to town. There were now several fistulas, proceeding from different orifices in the membranous portion of the urethra. From the anterior sinus forward, the urethra was totally impervious for the space of at least two inches—filled with a grisly callus. He was much reduced, and laboured under hectic, with copious night sweats.

Before proceeding to any operation, except to widen, externally, one of the fistulas, that the urine might descend with less interruption, I kept him thirteen days on a course of alteratives and tonics. The time was more than was requisite; but I had difficulty to determine in what manner I should operate, or whether I would operate at all. The negro's supplications at last determined me in the affirmative; and I proceeded to make an opening through the obliterated portion of the urethra, with a stilet. A silver catheter was then introduced into the bladder, and the fistulas were incised, the wound dressed, the silver catheter removed, and one of elm bark introduced into the bladder, and then kept fixed, until all was healed. On the 21st of June, he returned home in good health. A small fistula has since occurred, and remains, but does not increase, nor impair his health. This man never kept his bed one entire day after the operation; but walked the streets more or less every day during his stay in town, with the catheter in his urethra; from which he never, during the time, complained of either pain or uneasiness.

The catheter of elm bark is thus prepared. I take a thin strip of the inner bark, from one to one and a half inches wide, seasoned just so much as not to destroy its pliability, level the edges, and smear them with mucilage or glue; wrap the bark either spirally or longitudinally around a stilet, and roll with tape. The wire should be smeared thinly, but completely, with beeswax and tallow, to prevent its being retained by the glue. A good mechanic could make a nice article of this kind, which for the purpose of being left in the urethra, when a case may require such treatment, possesses great advantage over any other catheter; for when thus left, coated with mucilage, instead of acting as an irritant, it proves a fine emollient to the inflamed or lacerated parts.

*Fistula Lachrymalis.*—I have treated three cases of this disease with slippery elm bougies. In two of them, a slender probe could be passed, from the abscess, through the *ductus ad nasum*, into which bougies were introduced. Cures were effected in each case in about a fortnight.

The other case was that of a lady, whom I attended in the spring of 1833, at James River forge. She had laboured under the disease for several years. The duct was completely obliterated. I perforated the callus that filled it, with a sharp, triangular pointed probe, and inserted successive dried elm bougies, until it was dilated to the full natural size. I then kept it tented for four weeks, with bougies of the *fresh bark*, before the external opening was suffered to heal. The cure is perfect.

Several physicians in this neighbourhood have, on my recommendation, treated strictures with these bougies, and express themselves as well satisfied of their superiority as I have been.

*Calculus and the Operation of Lithontritie.*—There is no aspect in which I have ever considered, in a more favourable point of view, the benefit which is likely to result from the expanding property of the slippery elm bougie, than in relation to Civiale's operation of lithontritie.

I cannot but indulge the pleasing anticipation, that, with such assistance, the invention of that great benefactor of his race must eventually set aside the gorget and the staff, as a forlorn hope, to be retained only as a dernier resort, and not to be required to achieve a triumph over calculus once in a hundred cases.

The power of a lithontritor, from a half to three-fourths of an inch in diameter, upon calculus in the bladder, needs no description. It is easily conceived in idea; and yet it is no mere ideality, or idle creation of fancy, to believe that one of that size may be introduced. The urethra can be so expanded as to admit such a lithontritor. That it can, will be evident to any one who will duly consider the anatomy of the part, and the size of calculi that have passed through it. And, moreover, I have dilated it with bougies, to an extent exceeding the minimum of those measures, with but little effort, and without producing pain.

I have now in my possession a calculus, the greatest transverse diameter of which is six-tenths of an inch, the least, or that of the flat side five-tenths, the longitudinal eight-tenths, carefully measured, that passed spontaneously from a female patient in 1825, whilst using anti-lithics and diuretics; viz., aqua calcis, spts. nitre, and decoction of mallows. Arterial action was kept down by blood-letting. Sir A. Cooper extracted eighty through the urethra, with forceps. The urethra indeed seems constructed for expansion, almost as perfectly as the vagina; it is full of longitudinal folds or rugæ, which when simply unfolded, more than double its dimensions, even without drawing upon its elasticity, which is as great as that of any other part of the body; for we see it contracted into strictures, from slight irritation; and expanding into sacs, of astonishing dimensions, by the force of the urine, impelled only by the moderate contractile power of the bladder; and the division of the prostate gland into lobes, and its spongy texture, well adapt it for yielding to dilatation.

I think it difficult then to say, to what dimensions it would be unreasonable to suppose the adult urethra might be dilated, by the very gradual expansion of the bland emollient material here recommended.

Over half an inch would not, I can declare from experience; and it was with a view to be able to speak more experimentally, on this portion of my subject, that I so long delayed to communicate an improvement, that I consider so important in treating strictures, with the expectation, that I might encounter a case of calculus, that would justify a dilatation to the extent that the membrane was capable of bearing. But this is not the region for calculus, and I have neither met with, nor heard of a case, since commencing the use of this material for bougies.

The process of dilatation, with this substance, is calculated to prove beneficial, too, not merely in affording more room for action, but by lessening the irritability and sensibility of the parts, and thereby preparing the patient to endure a lithontritor for a much longer time, as well as of greater size; in illustration of which I will adduce a few facts. It is familiar to every one who has been under the necessity of introducing catheters or bougies, daily or oftener, that the first introduction is the most painful, and that each successive operation becomes less and less so, until at last nothing more than a pleasant titillation is experienced, but little, if any, greater in degree than was formerly experienced from the passage of the urine. And such is the case with every part of the body, as illustrated in the pituitary membrane, by the snuff-taker, in the retina, by the refiner and the glass-blower, and in the soles of the feet, by the pedestrian.

Independent of any effort of dilatation, we are informed, that Civiale always introduced a catheter or sound for several successive days previous to operating with the lithontritor, "to lessen irritability," and generally suffered it to remain in the urethra for several hours, and more especially, when the parts were unusually irritable. In one very irritable case, which he has related, the first introduction of the sound gave very great pain, but after operating with the lithontritor, so much had sensibility been diminished, that the passage of sand and gravel gave none.

A lady, upon whom I had performed the operation of lithotomy, in 1819, in Danville, Ky., had used anti-lithics, prescribed by quacks, by injection through the urethra—caustic alkali, among others—until the sensibility was so far impaired, that she was insensible to the contact of any thing that was applied to the part.

The surgical works and medical periodicals of latter years, abound in redoubtable obstacles to Civiale's operation; most of which have reference to the irritable state of the bladder aud urethra, from the long continued pressure of calculi. An answer to all of which I conceive to be comprised in my preceding remarks on irritability. Others are worthy of more particular consideration, among which, enlargement of the prostate gland is generally advanced, as constituting an insuperable obstacle. The difficulty of introducing the instrument here, seems to be the insurmountable part of the process. Now this *can* be surmounted by the elm bougie; but the question recurs, would it be advisable? In answer, I will venture as a surmise, that the pressure, from long continued efforts at dilatation, might chance to excite such a change of action in the part, as to effect a cure of the gland. A trial, surely, could not be amiss in a disease which is reckoned incurable. I have witnessed such results in indurated mammary glands, and why should I not in similar affections of the prostate? The effect of pressure made by bandages on external indurated parts is a familiar illustration.

Although I can admit no urethra to be too irritable, or too tender, no plurality of calculi too numerous, nor any stone to be too large for lithontritie, with a properly dilated urethra, yet there are cases wherein the lithotomist must come in aid. Patients who have stones that are encysted, I should not conceive to be safe subjects for lithontritie. But this is rare, and a surgeon who is master of his business can always detect it with the sound, before proceeding to any operation. And calculi sometimes, though still more rarely, adhere to the bladder. Morgagni gives an account of a large stone, with part of its surface thus attached. In the case of a gentleman on whom I performed lithotomy in Kentucky, a stone, the size of a hen's egg, adhered to the bladder with such firmness, that it was extracted with great difficulty. On coming away, about one-fourth of its anterior lamella was wanting, and was found attached to the bladder, so firmly that it required time and considerable force with the scoop and fingers to detach it. Such cases would be detected with a sound, or with the lithontritor, by turning the instrument once or twice round, after grasping the stone,—a precaution that should, at all events, and in every case, be observed, to gain assurance that no portion of the bladder was grasped with the stone, by the forceps.

Those two situations *alone* appear to me as exclusively requiring the operation of lithotomy. Other contra-indications that have been arrayed against lithontritie, such as diseased bladder, scirrhous prostate, severe affections of the kidneys, or of important vital organs, or calculus in very old men, with glairy urine, &c., are, I conceive, equally inauspicious to either, and every operation.

Some caution is necessary in using bougies or catheters of elm. Although this bark possesses a degree of tenacity surpassed by that of but few trees of the forest, yet when seasoned, and in a very dry state, it would be liable, in the hands of a careless, or awkward operator, to break off in the urethra or bladder. To obviate this danger, it should be immersed in water, for a longer period when it is very dry, which will restore tenacity to its outer fibres.—*Western Journal of Medical and Physical Sciences*, October, 1837.

